# PKCβII Activation Promotes Membrane-Proximal Enrichment of Ribosome-Bound RACK1

**DOI:** 10.3390/ijms27125310

**Published:** 2026-06-11

**Authors:** Ekaterina Shuvalova, Polina Fortygina, Gulnur Smirnova, Natialia Bal, Elena Alkalaeva, Peter Kolosov

**Affiliations:** 1 Engelhardt Institute of Molecular Biology, Russian Academy of Sciences, 119991 Moscow, Russia; 2 Institute of Higher Nervous Activity and Neurophysiology, Russian Academy of Sciences, 117485 Moscow, Russia

**Keywords:** RACK1, PKCβII, local translation, ribosome

## Abstract

The scaffold protein RACK1 (Receptor for Activated C Kinase 1) integrates signaling and translation, acting as a core component of the 40S ribosomal subunit. It binds activated Protein Kinase C (PKC) isoforms and membrane receptors. We used an auxin-inducible degron (AID2) system in human HAP1 cells to selectively deplete the free (cytoplasmic) pool of RACK1. The engineered RACK1–mAID–mClover3 fusion was rapidly degraded in the cytoplasm upon addition of 5-phenyl-indole-3-acetic acid (5-Ph-IAA), while the ribosome-bound pool remained detectable in ribosomal fractions, indicating that ribosome association makes RACK1 relatively less accessible to AID2-mediated proteolysis. Upon activation of PKCβII with phorbol-12-myristate-13-acetate (PMA), imaging at defined time points revealed closely matched kinetics of PKCβII membrane recruitment and membrane-proximal enrichment of ribosome-bound RACK1, peaking at ~10 min. Our data support a model in which activated PKCβII engages ribosome-bound RACK1 at membrane-proximal sites, consistent with a diffusion–capture mechanism in which PKCβII first accumulates at the membrane and then captures ribosome-bound RACK1, thereby recruiting the translational machinery to sites of signal input for membrane-proximal translation. These findings provide new insights into the spatial organization of translation.

## 1. Introduction

Receptor for Activated C Kinase 1 (RACK1) is a conserved WD40-repeat scaffold protein first identified for binding active PKCβII [1,2]. The seven-bladed β-propeller structure of RACK1 presents multiple protein–protein interaction surfaces, enabling it to bind kinases, G-proteins, phosphatases, and membrane receptors [1,3,4,5,6]. Consistent with its name, RACK1 interacts with activated PKC isoforms and is thought to stabilize their membrane association during signaling [1,3]. Remarkably, RACK1 is also an integral component of the 40S ribosomal subunit [7]. Cryo-EM studies place RACK1 on the head of the 40S near the mRNA exit channel [7], where it can recruit signaling proteins, including PKCβII, to the ribosome [8,9]. In this way, RACK1 provides a molecular interface linking extracellular signals to translational control: activated PKC associated with RACK1 can phosphorylate factors such as eIF6, thereby promoting translation [10]. Moreover, RACK1’s association with membrane receptors and cytoskeletal components suggests that it may facilitate the spatial organization of ribosomes at sites of local translation, such as focal adhesions or synapses [3,5,6].

Accumulating evidence indicates that the ribosome-bound form of RACK1 is critical for its stability and function. Gallo et al. demonstrated that dissociation of RACK1 from ribosomes markedly reduces its half-life and impairs global translation [11]. Conversely, ribosome-bound RACK1 persists and maintains translational capacity, indicating that the ribosome acts as a protective platform for RACK1. However, how RACK1 coordinates kinase signaling with translation in space and time remains unclear. In particular, it is unknown whether stimulus-induced redistribution of RACK1 reflects movement of ribosome-bound RACK1 or instead results from sequential recruitment to signaling sites.

To address this question, we applied the auxin-inducible degron (AID2) system [12,13] for rapid, conditional protein depletion. In the AID2 system, a mini-AID (mAID) tag is fused to the protein of interest, and the plant F-box protein OsTIR1(F74G) is expressed in mammalian cells. Addition of the auxin analog 5-phenyl-indole-3-acetic acid (5-Ph-IAA) induces OsTIR1(F74G) to bind the mAID tag, triggering ubiquitination and proteasomal degradation of the tagged protein [12]. This approach enables selective, acute removal of the free cytoplasmic pool of a protein while proteins embedded in large macromolecular assemblies, such as ribosomes, can be sterically protected from degradation. We generated HAP1 cells, a human near-haploid cell line, with endogenous RACK1 tagged with mAID-mClover3 and stably expressing OsTIR1(F74G). Using this system, we acutely depleted free RACK1 and analyzed the effects of PKC activation (by PMA) on its localization. To capture dynamic changes, we performed stimulus addition followed by fixation at defined time points and confocal microscopy imaging. This approach allowed us to compare the distribution of total RACK1 and ribosome-bound RACK1 between control and experimental conditions with good spatial resolution across defined stimulation time points.

## 2. Results

### 2.1. Generation of a RACK1-Degron Cell Line

**Figure 1:**
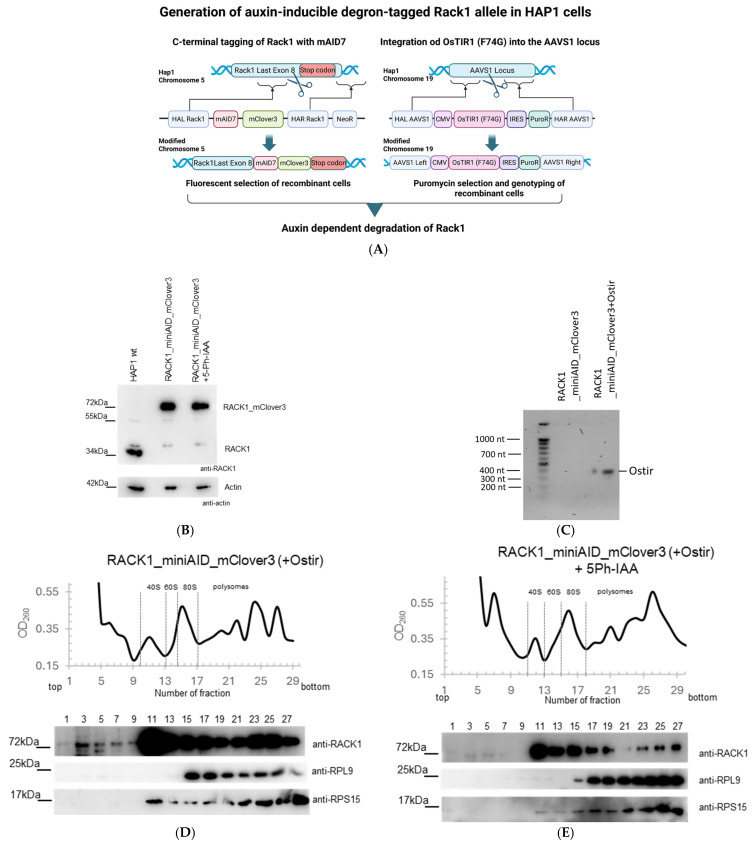
Ribosome-bound RACK1 is relatively less accessible to AID2-mediated degradation. (**A**) Strategy for endogenous RACK1 tagging with miniAID–mClover3 and stable OsTIR1(F74G) expression in HAP1 cells. (**B**) Western blot of parental (WT) and engineered HAP1 cell lysates in the presence or absence of auxin, probed for RACK1 and actin. Endogenous WT RACK1 is detected as an approximately 30 kDa band, whereas the tagged RACK1–mAID–mClover3 fusion is detected as an approximately 70 kDa band; the two species are separated by molecular weight and are shown in the same blot context for direct comparison. (**C**) PCR confirmation of OsTIR1(F74G) transgene integration. (**D**,**E**) Polysome profiles of HAP1 cells expressing RACK1–mAID–mClover3 and immunoblots showing the distribution of RACK1, RPS15, and RPL9 without (**D**) or with (**E**) auxin (5-Ph-IAA).

To study RACK1 function, we generated a HAP1-derived cell line with a degron tag inserted into the *RACK1* gene (Figure 1A). Using CRISPR/Cas9, a miniAID domain and the green fluorescent protein mClover3 were added to the C-terminus of RACK1. The gene for the plant F-box protein OsTIR1(F74G) was also stably introduced into this line, enabling auxin-inducible degradation of the target protein (following Natsume et al. [14]).

The resulting cell line expressed the mClover3 fluorescent protein. Western blot analysis revealed a single, higher molecular weight band (~70 kDa) corresponding to the RACK1-mAID-mClover3 fusion (length 633 aa), compared to the ~30 kDa (length 317 aa) band of native RACK1 in control HAP1 cells (Figure 1B). Genomic integration of the *OsTIR1* gene was confirmed by PCR with specific primers (Figure 1C) and puromycin selection.

### 2.2. Ribosome-Bound RACK1 Is Relatively Less Accessible to AID2-Mediated Degradation

After adding 5-Ph-IAA to induce degradation of the RACK1–mAID–mClover3 fusion, the green fluorescent signal and the corresponding band of the fusion protein did not completely disappear, indicating that a fraction of RACK1 remained. We reasoned that ribosome association may sterically hinder OsTIR1(F74G) from accessing the mAID tag on RACK1, consistent with previous observations that dissociated RACK1 is rapidly turned over [11]. To test this, we obtained polysome profiles from HAP1 cells expressing RACK1–mAID–mClover3 with and without 5-Ph-IAA treatment (Figure 1D) and determined RACK1 content in different fractions by immunoblotting.

Consistent with the known instability of free RACK1, only a small amount of the free form was detected in the top fractions of the sucrose gradient, which contain non-ribosomal proteins (Figure 1D). In contrast, significant amounts of RACK1–mAID–mClover3 were present in fractions corresponding to 40S subunits, 80S ribosomes and polysomes. After the addition of auxin, the free (non-ribosomal) pool of RACK1 was strongly reduced, whereas ribosome-bound RACK1 remained detectable in ribosomal fractions (Figure 1E). Together, these results support the conclusion that the ribosome-bound pool is relatively less accessible to AID2-mediated degradation compared with the non-ribosomal pool, supporting the idea that ribosomes can partially “shield” RACK1 from interaction with OsTIR1(F74G).

Lower molecular weight bands, observed in whole-cell lysates (Figure 1B) and in the fractions free of ribosomes (Figures S1–S4), likely represent non-specific products detected by the anti-RACK1 antibodies. These bands were neither degraded by auxin nor bound to ribosomes. Additional experiments with the proteasome inhibitor bortezomib and cycloheximide confirmed their persistent presence in cells, as well as the integrity of RACK1–mAID–mClover3 over different time intervals (0, 1, 2, 4, 6 h) (Figure S1), further supporting the non-specific nature of these bands.

### 2.3. Temporal Dynamics of PKCβII and RACK1 Translocation

**Figure 2:**
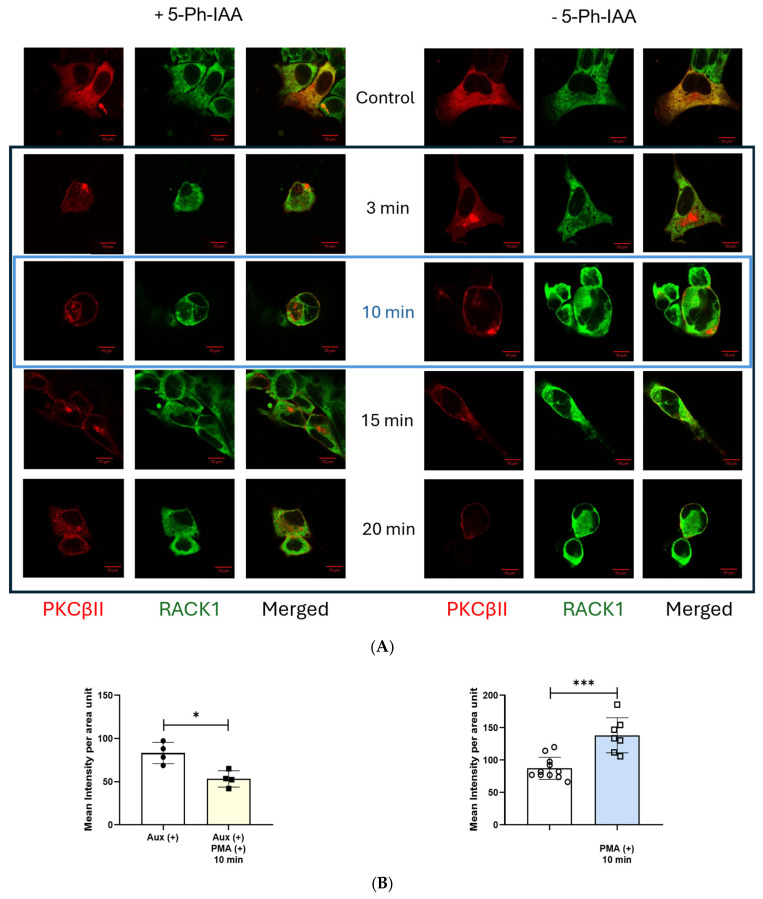
PKCβII activation promotes membrane-proximal enrichment of ribosome-bound RACK1. (**A**) Kinetics of PKCβII-mScarlet (red) and RACK1-mAID-mClover3 (green) in auxin-pretreated cells (left panel) and untreated cells (right panel) after PMA stimulation. (**B**) Mean fluorescence intensity of GFP–RACK1 normalized to cell area was quantified under the indicated treatment conditions. Auxin-treated control cells without PMA (Aux+/PMA−) compared with cells treated with auxin and stimulated with PMA for 10 min (Aux+/PMA+) (left panel). Intact control cells (Aux−/PMA−) compared with cells stimulated with PMA for 10 min in the absence of auxin (Aux−/PMA+) (right panel). Data are presented as mean ± SD, with individual data points shown. Statistical significance was assessed using the Mann–Whitney U test; * *p* < 0.05, *** *p* < 0.001.

We reasoned that the AID2 system would be useful for reducing the non-ribosomal pool of RACK1 that dissociates from ribosomes during experimental procedures. Therefore, we used this cell line to investigate the dynamics of ribosome-bound RACK1 and PKCβII following kinase activation with PMA (Figure 2A). Cells were transfected with a plasmid encoding a PKCβII-mScarlet fusion, such that red fluorescence reported PKCβII localization, while green fluorescence reported the localization of ribosome-bound RACK1 after auxin pretreatment to reduce the non-ribosomal signal.

After pre-treatment with 5-Ph-IAA to selectively deplete the more accessible non-ribosomal pool of RACK1, cells were stimulated with PMA and imaged over time. Although PMA activates multiple PKC isoforms, the analyses presented here specifically track the localization and kinetics of ectopically expressed PKCβII-mScarlet, and our conclusions are therefore restricted to this isoform. Upon PMA addition, we observed rapid redistribution of PKCβII to the plasma membrane. The PKCβII signal began accumulating at the membrane within 3 min and became predominantly membrane-associated by approximately 10 min (Figure 2A). From 15 min onward, PKCβII gradually redistributed back into the cytoplasm by 20 min. This behavior is consistent with a diffusion–capture mechanism, in which activated PKCβII rapidly associates with membrane lipids and anchoring partners, rather than undergoing directed transport.

Concurrently, the green fluorescent signal corresponding to ribosome-bound RACK1 became enriched in the membrane-proximal region. The increase in membrane-proximal RACK1 signal was detectable within 3–10 min of PMA stimulation and declined after 10–15 min. Importantly, the apparent temporal overlap between PKCβII membrane recruitment and RACK1 enrichment does not imply co-translocation of a preassembled PKCβII–ribosome complex. Instead, these dynamics are best explained by sequential diffusion and capture events: PKCβII first accumulates at the membrane upon activation, creating a transient membrane-localized interaction platform that subsequently captures ribosome-bound RACK1. Thus, rather than a stable cytosolic PKCβII–RACK1 complex moving as a unit, our data support a model in which activated PKCβII reaches the membrane independently and then engages RACK1 that remains associated with ribosomes. Additional representative cells imaged under the same treatment conditions using an independent confocal microscope showed a similar pattern of PKCβII membrane recruitment and membrane-proximal enrichment of RACK1–mAID–mClover3 (Figure S5).

In control cells lacking 5-Ph-IAA pre-treatment, PKCβII–mScarlet also redistributed to the plasma membrane upon PMA stimulation with kinetics similar to those observed in auxin-treated cells. A notable difference emerged at the 20-min time point: PKCβII remained membrane-associated in control cells but had largely redistributed back to the cytoplasm in auxin-treated cells. This observation is consistent with the notion that RACK1 stabilizes activated PKCβII at the membrane, thereby prolonging its residence time. In control cells, the presence of total RACK1, including ribosome-bound and residual non-ribosomal pools, likely contributes to this stabilizing interaction.

In contrast, the RACK1–mAID–mClover3 signal in control cells did not show pronounced membrane-proximal enrichment. We attribute this to the fact that, in these cells, the fluorescent signal reports total cellular RACK1–mAID–mClover3, including ribosome-bound RACK1 distributed throughout the cytoplasm. This substantial background likely masks the redistribution of the smaller membrane-proximal fraction, which becomes visible only when the non-ribosomal pool is selectively reduced using the AID2 system.

Quantitative analysis of mean green fluorescence intensity normalized to cell area (Figure 2B) is presented as a supportive measurement of changes in the overall RACK1–mAID–mClover3 signal. PMA stimulation led to a decrease in signal in auxin-treated cells but an apparent increase in control cells. This divergence is readily explained by differences in pool composition. In auxin-treated cells, the decrease may reflect redistribution of RACK1-positive ribosomal particles from the central cytoplasmic volume toward the cell periphery. In control cells, the measured increase likely reflects broader redistribution and accumulation of total RACK1 fluorescence near the cell periphery, including both broadly distributed ribosome-bound RACK1 and any residual non-ribosomal pool. However, we note that PMA-induced changes in cell shape can complicate fluorescence-based quantification, and the conclusion of membrane-proximal enrichment relies primarily on the time-course imaging shown in Figure 2A.

The imaging analysis was performed on individual cells with clearly detectable PKCβII–mScarlet expression, as HAP1 cells show relatively low transfection efficiency. Panoramic fields containing many non-transfected cells would not be informative for evaluating PKCβII-dependent redistribution of RACK1.

A comprehensive summary of the cell lines, treatment conditions, and the observed reporter behaviors (including those shown in Figure 2) is presented in Table 1.

## 3. Discussion

Taken together, the AID2-based imaging approach reveals a new facet of RACK1’s role in coupling signaling to translation. We show that ribosome-bound RACK1 is relatively less accessible to auxin-induced degradation than the non-ribosomal pool, consistent with earlier observations that ribosome association stabilizes RACK1 [11]. Importantly, a reduction in the non-ribosomal pool allowed us to more directly monitor the behavior of ribosome-bound RACK1. While PMA is a broad activator of PKC signaling, our study does not address the relative contributions of other endogenous PKC isoforms, focusing instead on the spatial dynamics of PKCβII. Under these conditions, ribosome-bound RACK1 becomes enriched at membrane-proximal sites following PKCβII activation, with kinetics that closely follow PKCβII membrane association. Crucially, this temporal correlation does not indicate that RACK1 transports PKCβII or vice versa. Instead, it indicates that activated PKCβII acts as a membrane-proximal capture site for ribosome-bound RACK1. This interpretation aligns with structural evidence showing that PKCβII binds RACK1 on the 40S ribosomal subunit [8], suggesting that the interaction occurs preferentially once PKCβII has reached the membrane.

This refined view updates the classical concept of RACK1 as a shuttling protein for activated PKCβII, which was originally inferred from stimulus-induced redistribution of the two proteins [15]. Our data support a revised model in which PKCβII defines a transient membrane platform that selectively recruits ribosome-bound RACK1, rather than being actively transported by it. In this way, PKCβII spatially organizes the translational machinery without requiring directed transport of ribosomes.

Importantly, the ribosome-bound state of RACK1 does not necessarily preclude its interaction with PKCβII. Although association with the 40S subunit may restrict some RACK1 interaction surfaces, structural and biochemical studies indicate that RACK1 can still serve as an accessible platform for signaling proteins in the ribosomal context. Thus, our data are consistent with a model in which activated PKCβII accumulates at membrane-proximal sites and subsequently engages ribosome-bound RACK1, without requiring cotransport of a preassembled cytosolic PKCβII–RACK1 complex.

This interpretation also reconciles our findings with earlier predictions that RACK1 promotes docking of ribosomes at specific membrane regions to enable local translation [7]. Rather than acting as a motor or carrier, RACK1 functions as a molecular interface that allows ribosomes to be captured at signaling hotspots. Interactions of RACK1 with membrane-associated cytoskeletal components, such as β-spectrin [16], may further stabilize these transient ribosome–membrane contacts, particularly at focal adhesions or synaptic sites, where on-demand rapid, localized protein synthesis is required [17,18]. Analogous diffusion–capture mechanisms have been described in other signaling contexts. For example, in T lymphocytes, RACK1 binds activated Lck and becomes enriched at the immunological synapse following TCR engagement [19]. These observations are consistent with a general role for RACK1 in coupling kinase activation to spatially restricted signaling outputs, rather than acting as a transport adaptor.

The functional consequence of recruiting ribosomes to the membrane is likely the selective local translation of specific mRNAs [20,21]. There is growing evidence that RACK1 regulates translation of defined transcript subsets [22,23]. For example, in neurons RACK1 modulates translation of synaptic mRNAs and interacts with FMRP (Fragile X mental retardation protein) to relieve repression of target mRNAs [22]. In astrocytes, RACK1 has been shown to bind certain transcripts (e.g., Kcnj10 encoding the Kir4.1 channel) and repress their translation until signaling cues trigger local derepression [24]. Thus, RACK1 may selectively facilitate the synthesis of proteins needed at signaling hubs. Membrane-localized activated PKCβII interacting with ribosome-bound RACK1 may establish spatially confined translation zones optimized for rapid translation of nearby transcripts encoding receptors, adhesion molecules, or cytoskeletal regulators, streamlining the local signaling response.

Ribosome heterogeneity and specialization. Our findings can be further understood in the broader context of ribosome heterogeneity. Emerging evidence indicates that ribosomes are not uniform but display specialized functions through differences in ribosomal protein composition, rRNA modifications, and association with specific translation factors or RNA-binding proteins [25,26,27]. RACK1 has been implicated in transcript-selective translation, including efficient translation of short mRNAs [28], and its association with distinct interaction partners may define ribosome subpopulations with specialized translational roles. Our data suggest that ribosome-bound RACK1 can be spatially enriched at membrane-proximal sites upon PKCβII activation, pointing to a mechanism by which signaling pathways can locally recruit a specific ribosomal subpopulation without requiring active transport of ribosomes as preassembled signaling complexes. Thus, RACK1 represents an additional layer of ribosome specialization that controls local translation at signaling hubs.

A recently developed ribosome-tethered degron strategy, termed Ribo-Tweezer, further highlights the importance of distinguishing between depletion of non-ribosomal RACK1 and direct depletion of RACK1 from mature ribosomes. In the Ribo-Tweezer system, Tir1 is tethered to mature ribosomes, enabling proximity-directed removal of selected ribosomal proteins, including RACK1, from ribosomal particles. This strategy is therefore conceptually different from our approach. Here, we used a standard AID2 configuration to reduce the more accessible non-ribosomal RACK1 pool while leaving a detectable ribosome-bound pool that could be followed during PKCβII activation. Notably, Chen et al. reported negligible unbound RACK1 in sucrose-gradient fractions and showed that ribosome-tethered Tir1 depleted ribosomal RACK1 more efficiently than a standard degron configuration [29]. These findings support our interpretation that free/non-ribosomal RACK1 may be difficult to detect in gradient fractions and that ribosome-bound RACK1 is relatively less accessible to standard AID2-mediated degradation. Thus, our system and Ribo-Tweezer address complementary questions: Ribo-Tweezer enables functional analysis of RACK1-depleted ribosomes, whereas our approach enriches the experimentally visible ribosome-bound RACK1 pool to study its stimulus-dependent spatial redistribution.

Our system further highlights the utility of the AID2 approach for studying compartment-specific translation. By preferentially reducing the more accessible non-ribosomal RACK1 while leaving a detectable ribosome-bound pool, we avoided the confounding effects of depleting the entire pool, which would be expected to perturb global translation, and instead enriched the experimentally visible ribosome-bound fraction. This partial and temporally controlled depletion of RACK1 provided an operational way to follow RACK1-positive ribosomal particles that remained detectable after 5-Ph-IAA treatment. Similar strategies could be applied to other ribosomal proteins or translation factors to study compartment-specific translation dynamics. For example, tagging and partially depleting a ribosomal protein might help identify ribosomes active in specific subcellular microdomains or stress conditions.

In conclusion, our data support a model in which RACK1 acts as a dynamic adaptor that links signal-activated PKCβII to the translational machinery via a diffusion–capture mechanism. We show that upon activation, PKCβII first accumulates at the membrane where it subsequently engages ribosome-bound RACK1, thereby spatially organizing translation. This mechanism provides a direct and flexible means by which extracellular signals can be converted into spatially organized protein synthesis without requiring active transport of ribosomes. Going forward, it will be interesting to determine which mRNAs are translated by these recruited ribosomes. Overall, our findings contribute to the emerging concept that translation is tightly coordinated with signaling localization in both space and time [7,16].

## 4. Materials and Methods

### 4.1. Cell Culture and Cell Lines

The human near-haploid HAP1 cell line (Horizon Discovery, Cambridge, UK) was cultured at 37 °C in a humidified atmosphere with 5% CO_2_ in Iscove’s Modified Dulbecco’s Medium (IMDM) supplemented with 10% fetal bovine serum (FBS), 2 mM GlutaMAX (all from Thermo Fisher Scientific, Waltham, MA, USA), and 50 U/mL penicillin/50 µg/mL streptomycin (Capricorn Scientific GmbH, Ebsdorfergrund, Germany). Cells were routinely passaged using 0.05% trypsin/EDTA upon reaching 70–80% confluency and were typically split at a 1:3 ratio. HAP1 cells were chosen because their near-haploid genetic background facilitates genome editing and clonal selection, which are critical for endogenous tagging of RACK1 and stable integration of OsTIR1(F74G). The near-haploid genome reduces allelic complexity and simplifies the isolation and validation of correctly modified clones. A limitation of this system is the relatively low transfection efficiency, which necessitated analysis of individual PKCβII-positive cells rather than panoramic fields.

### 4.2. Plasmids and Constructs

A donor plasmid for C-terminal tagging of the endogenous RACK1 locus (Gene ID: 10399) was constructed by cloning PCR-amplified left and right homology arms (HAL and HAR, ~500 bp each), flanking the stop codon, into the pMK289 vector (Addgene #72827) using SacI/XmaI and MluI restriction sites (Addgene, Inc., Watertown, MA, USA). The resulting plasmid enables in-frame insertion of a miniIAA7 (mAID) degron, mClover, and a neomycin resistance (NeoR) cassette.

For CRISPR/Cas9-mediated targeting, sgRNA sequences designed to cut near the RACK1 stop codon were cloned into the pX458 vector (Addgene #48138), which co-expresses Cas9, sgRNA, and mScarlet.

The orthogonal auxin receptor OsTIR1(F74G) was expressed from the pMK232 plasmid (Addgene #72834), containing a CMV-driven OsTIR1(F74G)-IRES-PuroR cassette flanked by AAVS1 homology arms. A specific sgRNA targeting the AAVS1 locus was also cloned into the pX458 vector. All primer and sgRNA sequences are listed in Table 2.

### 4.3. Generation of HAP1 Cell Lines with an Auxin-Inducible Degron Tag on RACK1

The HAP1 degron line was generated in two sequential steps.

Step 1: Endogenous tagging of RACK1. HAP1 cells were transfected in a 10 cm dish with Lipofectamine 3000 (Invitrogen, Carlsbad, CA, USA) using a 1:2 mass ratio of the RACK1-targeting pX458-sgRNA vector to the pMK289-based donor plasmid (total 5 µg DNA). After several days, individual mClover-positive colonies were manually picked, expanded, and validated by genomic PCR, sequencing, and Western blotting to confirm RACK1 tagging in the genome.

Step 2: Integration of OsTIR1(F74G). A validated RACK1–mAID–mClover3 clone was transfected with a 1:1 mixture of the AAVS1-targeting pX458-sgRNA vector and the pMK232 donor plasmid (total 4 µg DNA) using Lipofectamine 3000. Puromycin selection (1 µg/mL) was applied 48 h post-transfection. Resistant cells were plated at low density to obtain single-cell clones, which were screened by junctional PCR and sequencing to confirm homozygous integration of the OsTIR1(F74G) cassette into the AAVS1 locus.

### 4.4. Western Blotting

Protein extraction and sample preparation. For protein detection, cells were washed with PBS and lysed in RIPA buffer (50 mM Tris-HCl pH 8, 150 mM NaCl, 1% Triton X-100, 0.5% sodium deoxycholate, 0.1% SDS) supplemented with a protease inhibitor cocktail (cOmplete™, Mini, EDTA-free Protease Inhibitor Cocktail (Roche, Basel, Switzerland)). Protein concentration was determined using the Pierce BCA Protein Assay Kit (Thermo Fisher Scientific). Equal amounts of protein (20 µg) were used for subsequent analysis. Proteins were resolved by standard SDS-PAGE on 12% polyacrylamide gels and transferred to PVDF membranes (Bio-Rad, Hercules, CA, USA) using a semi-dry transfer system at a constant current of 250 mA for 1 h. All membranes were blocked for 15 min in EveryBlot Blocking Buffer (Bio-Rad, #12010020) and then incubated overnight at 4 °C with primary antibodies diluted in the same buffer. The following primary antibodies were used: rabbit anti-RACK1 (Servicebio, Wuhan, China, #GB11909-100, 1:1000) and rabbit anti-RPL9 (Abcam, Cambridge, UK, ab182556) and anti-RPS15 (Servicebio, #GB111967-100, 1:1000). Following washes, membranes were incubated for 1 h at room temperature with HRP-conjugated secondary antibodies (Bio-Rad, anti-rabbit #170-6515, 1:3000). Signal was detected using an enhanced chemiluminescence (ECL) substrate and visualized on a ChemiDoc imaging system (Bio-Rad).

### 4.5. Polysome Profile Analysis

HAP1 cells were grown in a 10 cm dish to about 70% confluency. For degradation of RACK1–mAID–mClover3, cells were treated with 5-Ph-IAA (500 nM) for 3 h. Prior to lysis, cells were treated with 100 µg/mL cycloheximide for 15 min. The cells were then harvested, washed twice with PBS containing 100 µg/mL cycloheximide, and centrifuged at 200× *g* for 5 min at 4 °C. The pellet was lysed in 400 µL of ice-cold polysome lysis buffer (20 mM Tris-HCl, pH 7.5, 140 mM KCl, 5 mM MgCl_2_, 1 mM DTT, 1% (*v*/*v*) Triton X-100, 0.1 mg/mL cycloheximide, protease inhibitor cocktail (Promega, Madison, WI, USA), and 100 U/mL RNase inhibitor RiboLock, Thermo Scientific) for 10 min on ice, followed by centrifugation at 16,000× *g* for 20 min at 4 °C.

The supernatant was loaded onto a 12 mL linear 7–50% (*w*/*v*) sucrose gradient (20 mM Tris-HCl, pH 7.5, 140 mM KCl, 5 mM MgCl_2_, 1 mM DTT) and centrifuged at 35,000 rpm in an SW41Ti rotor (Beckman, Brea, CA, USA) at 4 °C for 3 h. The gradient was then fractionated into 200 µL fractions while monitoring absorbance at 260 nm. For Western blot analysis, 7 µL of every second fraction was separated by 10% SDS-PAGE and transferred for immunoblotting. Antibodies against RPL9 (Abcam, ab182556), RPS15 (Abcam) and RACK1 (Servicebio) were used for detection. The distribution of RACK1 across these fractions was assessed by Western blotting to determine the proportion of RACK1 associated with polysomes or ribosomal subunits versus the free cytoplasmic pool.

### 4.6. Fixed-Cell Imaging and PKCβII Translocation Assay

HAP1 RACK1–mAID–mClover3 cells were seeded on glass coverslips and transfected with the PKCβII-mScarlet plasmid. To deplete the non-ribosomal RACK1 pool, cells were treated with 500 nM 5-Ph-IAA for 3 h. PKCβII was activated by adding 1 µM PMA. Cells were fixed at specified time points (0, 3, 10, 15, 20 min post-stimulation) with 4% paraformaldehyde. Fixed samples were mounted and imaged using a Thorlabs confocal LS microscope with a 63× oil-immersion objective at 488 nm/561 nm (Thorlabs, Newton, NJ, USA). Imaging analysis was performed on individual cells with clearly detectable PKCβII–mScarlet expression, as HAP1 cells show relatively low transfection efficiency; panoramic fields containing many non-transfected cells would not be informative for evaluating PKCβII-dependent redistribution of RACK1. Additional representative images shown in Figure S5 were acquired under the same treatment conditions using an independent Nikon Eclipse Ti2 confocal microscope equipped with an Apo TIRF 60×/1.49 oil objective (Tokyo, Japan). These images were used only as qualitative examples and were not included in the quantitative fluorescence intensity analysis shown in Figure 2B.

## Figures and Tables

**Table 1:** Summary of cell lines, treatment conditions, and reporter behavior.

Cell Line/Derivative	Genetic Modification	Treatment	Observed/Reporter Behavior of RACK1–mAID–mClover3	Observed/Reporter Behavior of PKCβII–mScarlet (If Expressed)
Parental HAP1	None (wild-type)	–	No fluorescence; ~30 kDa endogenous RACK1	–
RACK1–mAID–mClover3	Endogenous RACK1 C-terminally tagged with mAID–mClover3	–	Green fluorescence; ~70 kDa fusion protein; ribosome-bound and non-ribosomal pools present	–
RACK1–mAID–mClover3 + OsTIR1(F74G)	Same as above + stable integration of OsTIR1(F74G)	–	Same as above; OsTIR1 expressed but inactive	–
RACK1–mAID–mClover3 + OsTIR1(F74G)	Same	+5-Ph-IAA (3 h)	Non-ribosomal pool strongly reduced; ribosome-bound pool remains detectable (relative protection)	–
RACK1–mAID–mClover3 + OsTIR1(F74G) + transient PKCβII–mScarlet	Same	–	Green fluorescence (total RACK1)	Red fluorescence (cytoplasmic)
Same	Same	+5-Ph-IAA, then + PMA (10 min)	Green fluorescence enriched at membrane-proximal region (ribosome-bound pool)	Red fluorescence translocated to plasma membrane
Same	Same	+PMA (no auxin)	No clear membrane enrichment (total RACK1 signal masks redistribution)	Red fluorescence translocated to plasma membrane (prolonged residence)

**Table 2:** Primers used in this study.

Primers	Sequences
gRNA_ostir2_f	CAC CGG GGC CAC TAG GGA CAG GAT
gRNA_ostir2_r	AAA CAT CCT GTC CCT AGT GGC CCC
gRNA_Rack1_f	CAC CTG GCA CAC GCT AGA AGT TTA
gRNA_Rack1_r	AAA CTA AAC TTC TAG CGT GTG CCA
HAL_Rack1_f	TTT GAG CTC AGC CAG GTT GAT GAG GAT GCC CTC G
HAL_Rack1_r	TTT CCC GGG CGT GTG CCA ATG GTC ACC TGC
Rack1_HAR_f	CCC ACG CGT AAG TTT ATG GCA GAG CTT TAC AAA TAA A
Rack1_HAR_r	TTT ACG CGT GAC CAT GTT GAC AGA GAC TGC TAA CAT G
Rack1_check_f	CCT CTC AGG TGA TTC TGC TTC CAG
mAID-check_r	ATC TTT AGG ACA AGC ACT CTT CTC C
Ostir2_check_f	TAG CGC TGC CAC CAT GAC ATA CTT TCC
Ostir2_check_r	CCA GCA GCT CCA GAC TTT CGT CAG A

## Data Availability

The original contributions presented in this study are included in the article and Supplementary Materials. Further inquiries can be directed to the corresponding author.

## Supplementary Materials

The following supporting information can be downloaded at: https://www.mdpi.com/article/10.3390/ijms27125310/s1.

## Abbreviations

The following abbreviations are used in this manuscript:

RACK1	Receptor for Activated C Kinase 1
PKC	Protein Kinase C
PKCβII	Protein Kinase C βII
PMA	Phorbol 12-Myristate 13-Acetate
5-Ph-IAA	5-Phenyl-indole-3-acetic acid
