# Supplementary material for: PKCβII Activation Promotes Membrane-Proximal Enrichment of Ribosome-Bound RACK1
Source: Int J Mol Sci. 2026 Jun 11;27(12):5310. doi: 10.3390/ijms27125310 (PMC13300571; doi:10.3390/ijms27125310)
Supplement: Supplementary file 1 [file ijms-27-05310-s001.zip › ijms-4305594-supplementary.pdf]

Supplementary figures legends

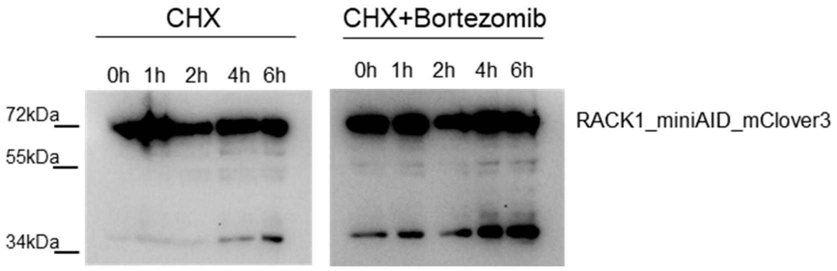

Figure S1. Accumulation of non-specific/degradation products of Rack1_miniAID_mClover3 in Hap1 lysate in the presence of cycloheximide (CHX) on the left and in the presence of cycloheximide and bortozomib (a 26S proteasome inhibitor) on the right.

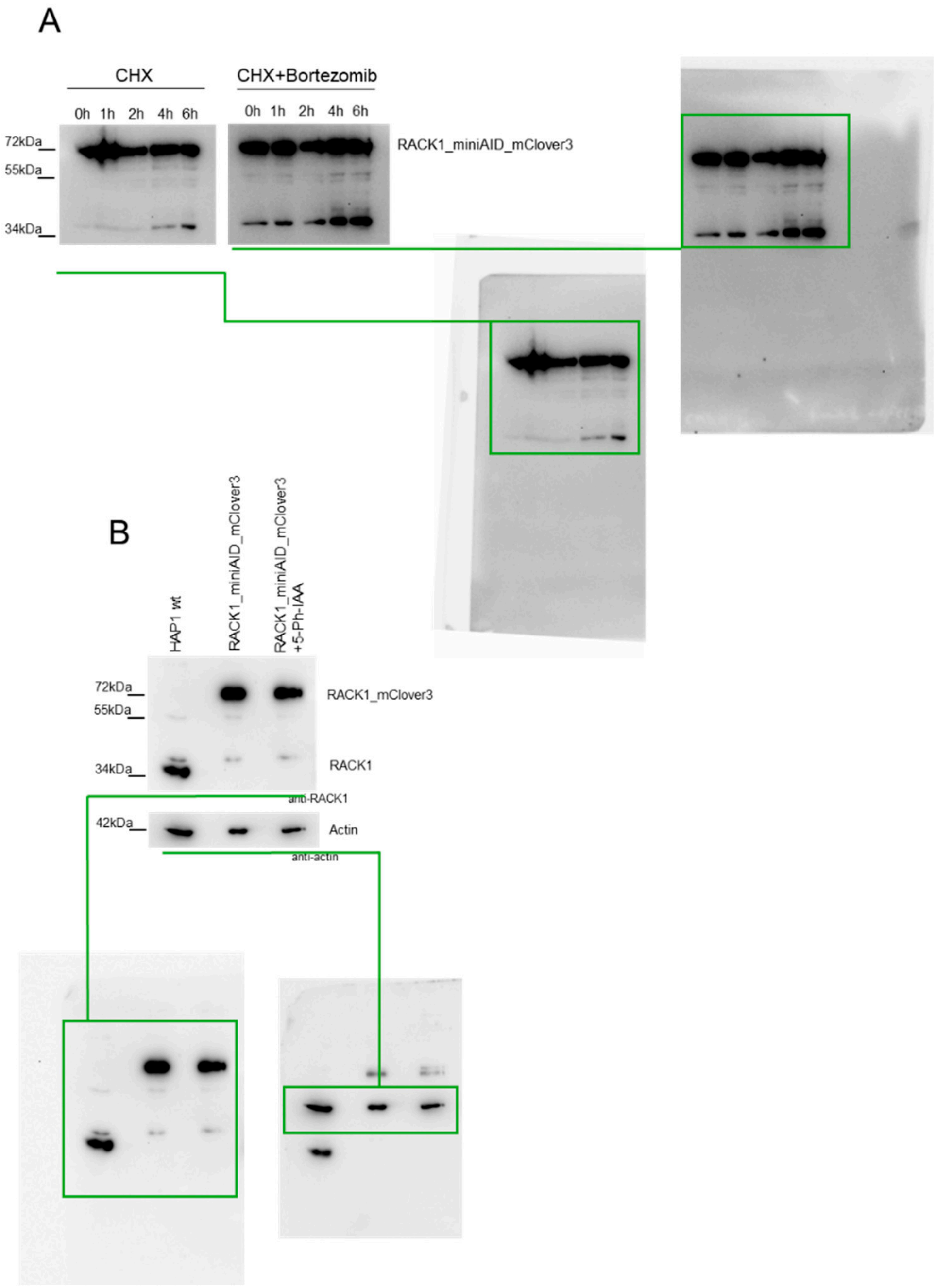

Figure S2. Raw data of western blots.

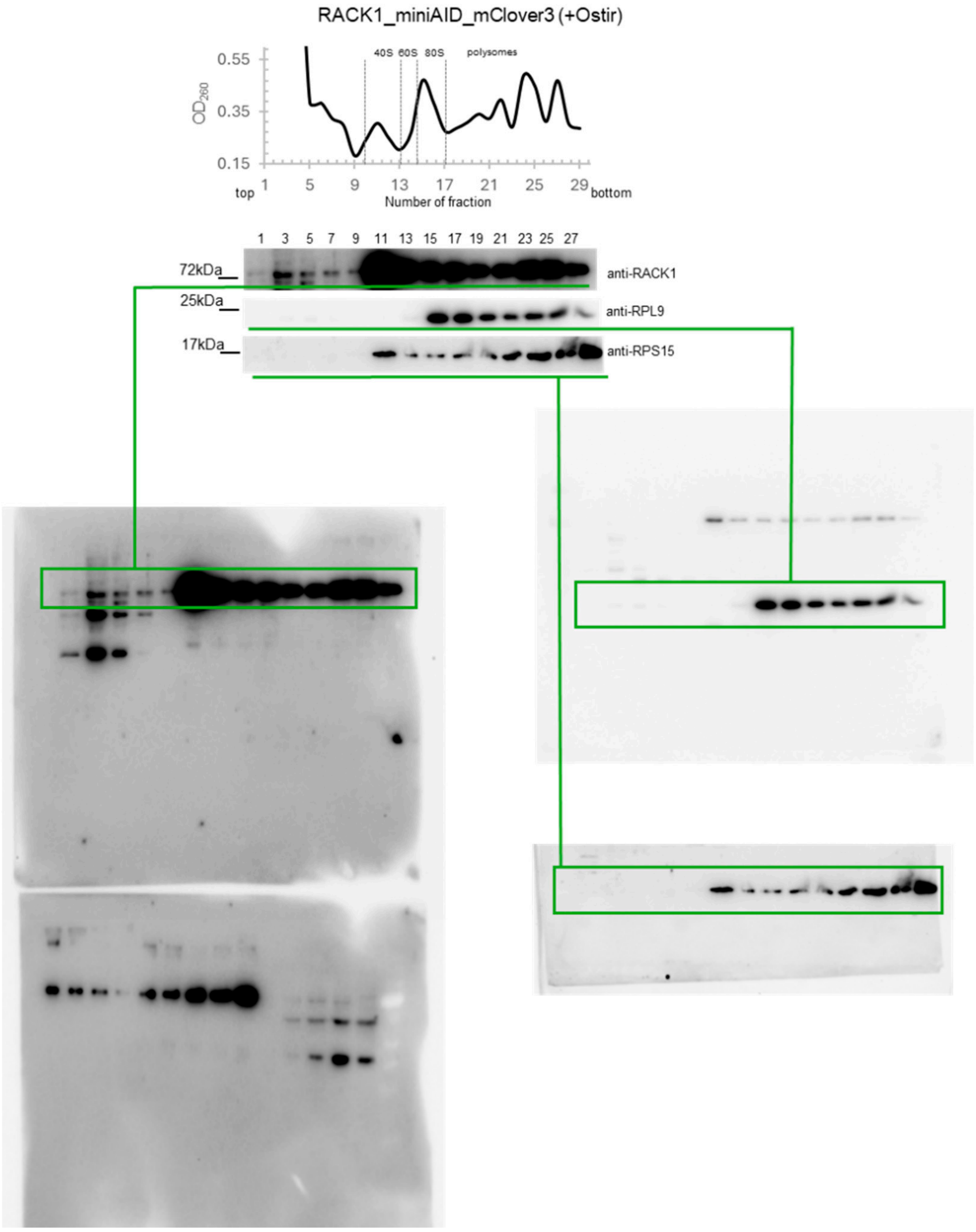

Figure S3. Raw data of western blots.

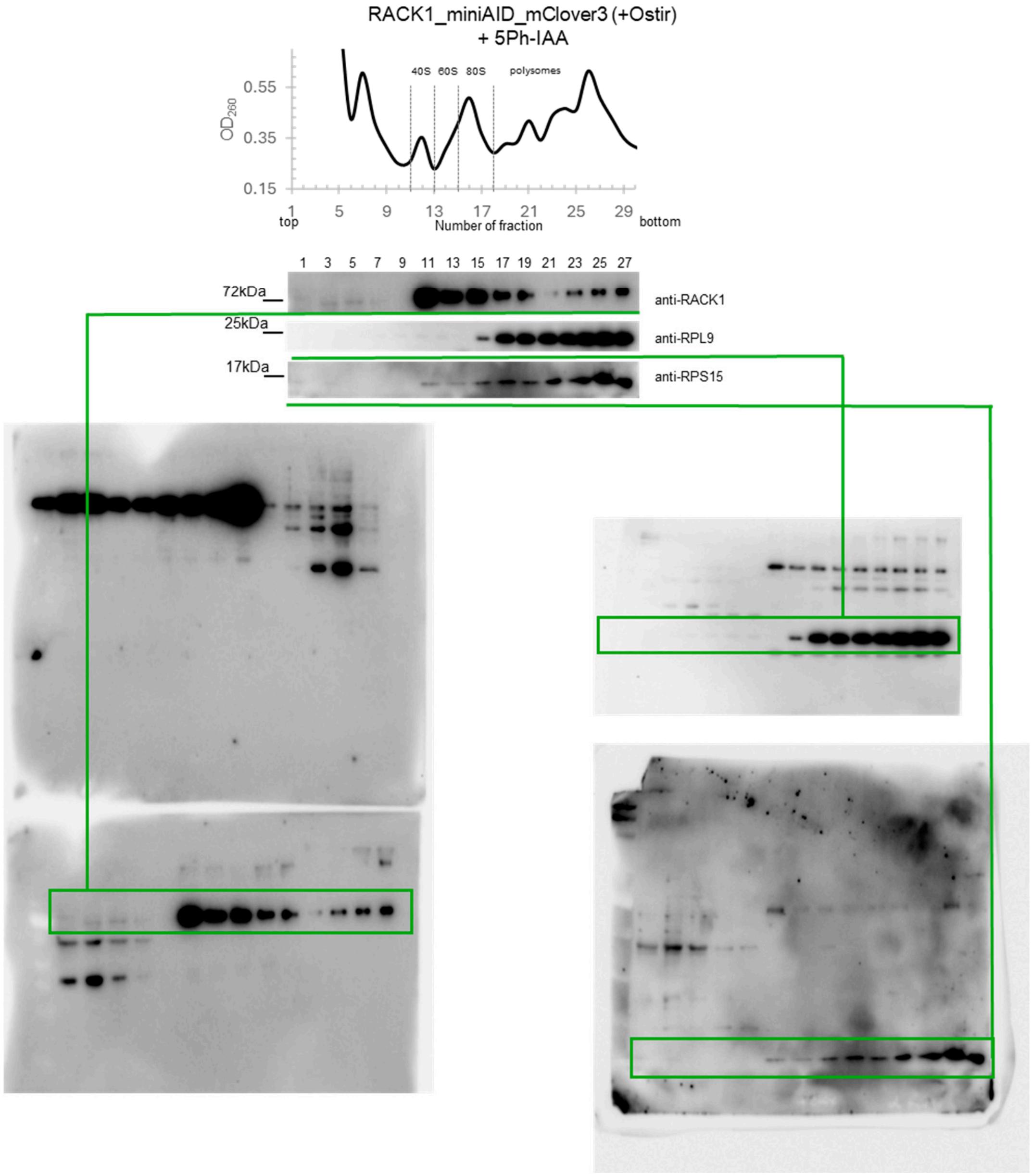

Figure S4. Raw data of western blots.

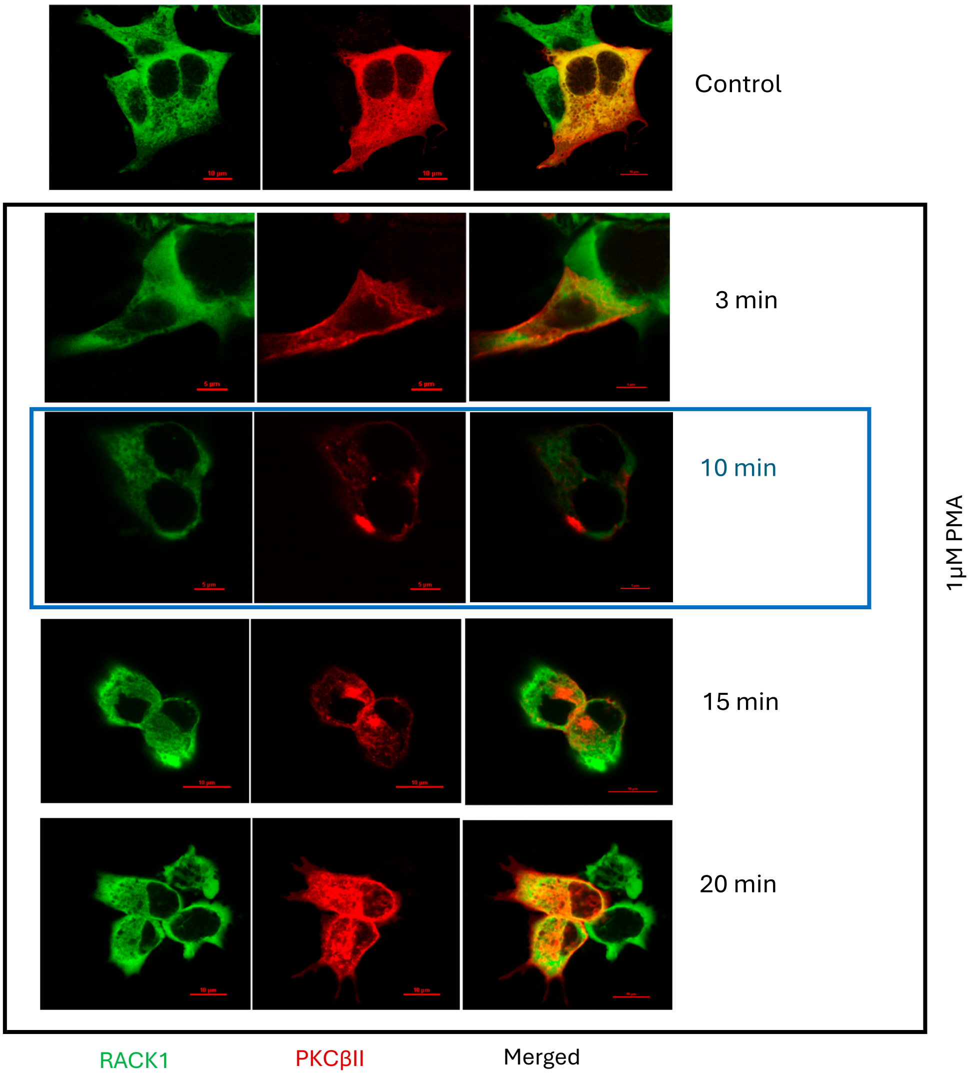

Figure S5. Additional representative images of PKCβII–mScarlet and RACK1–mAID–mClover3 redistribution after PMA stimulation. Representative confocal images of additional PKCβII–mScarlet-positive HAP1 RACK1–mAID–mClover3 cells treated under the same experimental conditions as in Figure 2A. Red fluorescence corresponds to PKCβII–mScarlet, and green fluorescence corresponds to RACK1–mAID–mClover3. Images were acquired using an independent Nikon Eclipse Ti2 confocal microscope equipped with an Apo TIRF 60×/1.49 oil objective and are shown as additional qualitative examples, not as part of the quantitative fluorescence analysis in Figure 2B. The images illustrate a similar pattern of PKCβII membrane recruitment and membrane-proximal enrichment of RACK1–mAID–mClover3 after PMA stimulation. Scale bars, 5 µm for the 3 and 10 min panels and 10 µm for the other panels.
